# The *AfldrnA* Transcription Factor Is a Pivotal Regulator of the Conidiation–Sclerotial Formation Balance in *Aspergillus flavus*

**DOI:** 10.3390/jof12040277

**Published:** 2026-04-14

**Authors:** Mohammed A. Abdo-Elgabbar, Bashir Salim, Sang-Cheol Jun, Yu-Kyung Kim, Saeed Alasmari, Kap-Hoon Han

**Affiliations:** 1 Department of Pharmaceutical Engineering, Woosuk University, Wanju 565-701, Republic of Korea; mohsudan0@gmail.com (M.A.A.-E.); scjun@woosuk.ac.kr (S.-C.J.); yg6715@woosuk.ac.kr (Y.-K.K.); 2 Camel Research Center, King Faisal University, Al-Ahsa 31982, Saudi Arabia; bsalim@kfu.edu.sa; 3 Department of Biology, Faculty of Arts and Sciences, Najran University, Najran 1988, Saudi Arabia; smalasmari@nu.edu.sa

**Keywords:** *AfldrnA* gene, asexual development, *Aspergillus flavus*, conidiation, gene deletion, helix–loop–helix (HLH) transcription factor

## Abstract

*Aspergillus flavus* is a globally distributed filamentous fungus of major agricultural and medical importance, capable of producing carcinogenic aflatoxins and forming two specialized developmental structures, conidia and sclerotia. While the molecular framework governing conidiation has been well characterized in *Aspergillus nidulans*, the corresponding mechanisms in *A. flavus* remain somewhat unelucidated. In this study, we identified and functionally characterized *AfldrnA*, a gene encoding a basic helix–loop–helix (bHLH) transcription factor. Targeted deletion of *AfldrnA* resulted in an aconidial phenotype accompanied by a significant increase in sclerotia formation, whereas complementation with the intact *AfldrnA* gene restored conidiation and reduced sclerotia development. Phenotypic assays revealed that the Δ*AfldrnA* mutant exhibited normal vegetative growth, unchanged antifungal susceptibility, and unaffected aflatoxin B_1_ production, indicating that *AfldrnA* primarily regulates developmental rather than metabolic differentiation. Additionally, observed differences between standard and dark incubation conditions suggest that *AfldrnA* may be involved in environmentally responsive regulation of fungal development. Overall, this study identifies *AfldrnA* as a pivotal transcriptional regulator essential for coordinating conidiation and sclerotia formation in *A. flavus*, providing new insights into the genetic and environmental regulation of fungal developmental programs.

## 1. Introduction

*A. flavus* is a globally distributed fungus with major agricultural, biotechnological, and public health relevance. It produces aflatoxins, potent carcinogenic secondary metabolites that pose significant risks to crops, livestock, and human health [1,2,3,4,5].

*A. flavus* reproduces both sexually and asexually. Asexual reproduction primarily involves the formation of conidia, which plays a crucial role in fungal dispersal and environmental persistence. The fungus also produces sclerotia, which serve as survival structures under adverse conditions and are thought to represent degenerated sexual structures [6,7]. These features enable *A. flavus* to thrive in diverse habitats, particularly in warm and humid regions, with optimal growth temperatures ranging between 12 and 48 °C [8,9].

The molecular control of conidiation in *A. nidulans*, a well-established model for fungal development, is governed by a hierarchical gene network comprising *brlA*, *abaA*, and *wetA*, which are expressed sequentially during conidiation [10,11]. Among these, *brlA* acts as a master transcription factor initiating conidiophore development and coordinating both asexual and sexual reproductive pathways [10,12]. Upstream regulatory genes such as *fluG, flbA* are critical for proper *brlA* expression and the initiation of conidiation [13,14]. Mutations in these genes can disrupt conidiation process and markedly affect secondary metabolite biosynthesis, including aflatoxin production [15]. In contrast, the genetic mechanisms governing conidiation and sclerotia formation in *A. flavus* remain less clearly defined. Transcription factors such as NsdC and NsdD have been implicated in both processes, influencing conidiophore architecture and aflatoxin biosynthesis [4]. Moreover, LaeA, a global regulator of secondary metabolism in *Aspergillus* species, plays an essential role in aflatoxin production and in the regulation of conidiation and sclerotia development in *A. flavus* [16].

In this study, we investigated the function of the *drnA* gene in *A. flavus* (*AfldrnA*). Previous research in *A. fumigatus* demonstrated that *AfudrnA* is vital for conidiation and for the regulation of *brlA* expression [17]. Here, we characterize the orthologous gene in *A. flavus* and evaluate its role in conidiation and sclerotia development. Understanding the functional contribution of *drnA* in *A. flavus* will enhance our knowledge of the genetic control of fungal development and secondary metabolism in this economically and medically important species.

## 2. Materials and Methods

### 2.1. Identification of the AfldrnA Gene

The *AfldrnA* locus, encoding a helix–loop–helix (HLH) domain-containing protein, was previously characterized in *A. nidulans* [18]. To identify its ortholog in the *A. flavus* genome, the open reading frame (ORF) of the *A. nidulans drnA*-ortholog (locus ANID_04394.1) was used as a query for BLASTP analysis against the Broad Institute database (http://www.broadinstitute.org). The search identified AFL2G_04716 as the *drnA* gene in *A. flavus* (*AfldrnA*). Additionally, the *A. oryzae ecdR* gene and *A. fumigatus drnA* (*AfudrnA*) were recognized as corresponding orthologs.

### 2.2. Strains and Media

The *A. flavus* strains used in this study included NRRL3357 (wild type), NRRL3357.5, and NRRL3357.10. NRRL3357 was obtained from the U.S. Department of Agriculture (USDA). NRRL3357.5 was derived as a *pyrG* mutant, and NRRL3357.10 was generated by reintroducing the *pyrG* gene into NRRL3357.5 and served as an isogenic control (Table 1) [19]. Standard transformation protocol was applied as described for *A. nidulans* [20]. Minimal medium (MM) was prepared following the method of [21,22,23], while complete medium (CM) was produced by supplementing MM with 1.5 g yeast extract, 1.5 g casein hydrolysate, and 10 mL vitamin solution per liter. Uracil (0.12%) and uridine (0.12%) were added to the media when required to support the growth of *pyrG* auxotrophic strains (NRRL3357.5 and complemented strains), which are unable to grow without supplementation, thereby ensuring consistent growth conditions across all strains. Cultures were incubated at 30 °C under standard laboratory conditions without controlled light exposure; thus, no defined light intensity or photoperiod was applied. Exposure to light was limited to brief, incidental illumination during routine handling. For dark treatment, culture plates were completely wrapped in aluminum foil to prevent any light exposure throughout the incubation period.

### 2.3. Construction of AfldrnA Deletion and Complementation Strains

**Figure 1:**
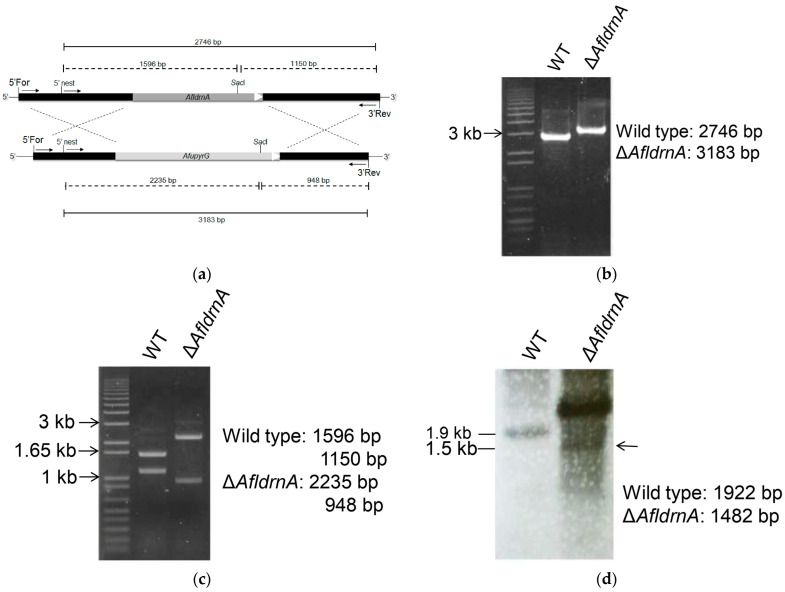
Construction of *AfldrnA* gene deletion. (**a**) Schematic representation of the *AfldrnA* deletion construct. Approximately 0.6 kb of upstream and downstream flanking regions were fused at the 5′ and 3′ ends of the deletion cassette. Solid lines indicate the sizes of diagnostic PCR amplicons, whereas dashed lines indicate restriction enzyme digestion fragments. 5′ nest and 3′ Rev represent primers used for diagnostic PCR; (**b**) PCR screening using primer pairs *AfldrnA* 5′ nest and *AfldrnA* 3′ Rev. The wild type yielded a 2.7 kb fragment, whereas the deletion mutant generated a 3.2 kb amplicon; (**c**) *Sac*I digestion of PCR products produced fragments of approximately 1.6 and 1.2 kb for the wild type, and 2.2 and 0.9 kb for the deletion mutant; (**d**) Southern blot confirmation of *AfldrnA* deletion. Genomic DNA was double-digested with *Hind*III and *Xma*I and subsequently hybridized with a 0.7 kb digoxigenin (DIG)-labeled probe generated by PCR amplification of the 3′ flanking region of *AfldrnA* gene using primers *AfldrnA* 3′ For *pyrG* tail and *AfldrnA* 3′ Rev. The expected hybridization signals were 1.9 kb for the wild type and 1.5 kb for the deletion mutant (indicated by arrow). The strong band observed in the deletion mutant may be due to incomplete enzymatic digestion of genomic DNA, possibly resulting from a high gDNA concentration.

The *AfldrnA* gene deletion was carried out using the *A. fumigatus pyrG* (*AfupyrG*) gene as a selectable marker through the double-joint PCR method [24]. Approximately 0.6 kb of the upstream and downstream flanking regions of *AfldrnA* were amplified and fused with *AfupyrG* to construct the replacement cassette (Figure 1a). Polyethylene glycol (PEG)-mediated protoplast transformation was carried out according to established methods [20]. 

For gene complementation, a 2.5 kb fragment containing the *AfldrnA* open reading frame (1.2 kb), promoter (1.1 kb), and terminator (0.2 kb) regions was amplified using the primer pairs *AfldrnA* Complementary For-*Asc*I and *AfldrnA* Complementary Rev-*Sma*I (Table 2), as well as Pfu-X polymerase (SolGent™ Pfu-X DNA Polymerase, Cat. No. SPX16-R250, Daejeon, South Korea). The PCR product was digested with *Asc*I and *Sma*I and ligated into the similarly digested pPTRI-PA vector, a modified pPTRI chromosomal integration vector carrying a pyrithiamine resistance gene (TaKaRa, Code No. 3621, Dalian, China), using T4 DNA ligase (Invitrogen, Cat. No. 15224-017, Carlsbad, CA, USA) (Figure S1). Transformants were selected on MM supplemented with 0.6 M KCl, 0.12% uracil, 0.12% uridine, and 0.1 μg/mL pyrithiamine to isolate *AfldrnA*-complemented strains.

### 2.4. Southern Blot Hybridization Analysis

The deletion of *AfldrnA* was verified by Southern blot analysis. Genomic DNA was extracted using the DNeasy Plant Mini Kit (Qiagen, Cat. No. 69104, Hilden, Germany) and double-digested with *Hind*III and *Xma*I. Digested fragments were separated by agarose gel electrophoresis and transferred to a Qiabrane positively charged nylon membrane (Qiagen, Cat. No. 61010, Hilden, Germany). Hybridization followed the DIG application manual (Roche Diagnostics GmbH, 2008). A 0.7 kb DIG-labeled probe was generated using the DIG High Prime DNA Labeling and Detection Starter Kit II (Roche, Cat. No. 11585614910, Mannheim, Germany). Hybridization and detection were performed according to the manufacturer’s instructions to confirm targeted deletion of *AfldrnA*.

### 2.5. Examination of Aflatoxin B_1_ Production by Thin Layer Chromatography (TLC)

Aflatoxin B_1_ production was examined using thin-layer chromatography (TLC) as described in [16] with slight modifications. *A. flavus* NRRL3357.5, *AfldrnA*-deletion, and *AfldrnA*-complemented strains were point-inoculated onto Potato Dextrose Agar (PDA) supplemented with uracil and uridine and incubated at 30 °C for seven days. Two plugs from each culture were transferred to 1.5 mL microcentrifuge tubes, and aflatoxins were extracted with 500 μL chloroform by incubation for 1 h. Samples were centrifuged at 12,000 rpm for 5 min, and 400 μL of the supernatant was collected. The solvent was evaporated in a 65 °C water bath, and residues were resuspended in 20 μL chloroform. For TLC, 10 μL of each sample was spotted onto silica gel plates (Merck KGaA, Silica gel 60 F254, Cat. No. HX391037, Darmstadt, Germany) and developed with Toluene:Methanol:Acetic acid (80:15:5, *v*/*v*/*v*) as the solvent system. Aflatoxin B_1_ spots were visualized under UV light.

### 2.6. Conidial and Sclerotia Quantification

Cultures were grown under the indicated conditions. For conidial quantification, conidia were collected from each colony and suspended in sterile distilled water containing 0.01% Tween 80. The suspension was thoroughly mixed and diluted appropriately (typically 10–100-fold) to obtain a countable concentration (10^−3^). Conidia were counted using a hemocytometer, with each measurement performed in triplicate and the mean value reported. For sclerotial quantification, sclerotia were counted manually per plate under a stereomicroscope. Each count was performed in triplicate, and the mean value was calculated. All quantifications were performed under standardized sampling and incubation conditions.

### 2.7. Software

Bioinformatic analyses and sequence design were performed using multiple software packages. LaserGene (DNASTAR, Madison, WI, USA) was primarily used for constructs designing. LaserGene and ChromasPro (Technelysium Pty Ltd, South Brisbane, QLD, Australia) were used for sequence assembly and visualization. Multiple sequence alignments were conducted using ClustalW (European Molecular Biology Laboratory–European Bioinformatics Institute, Hinxton, Cambridge, UK). Primer design was performed using LaserGene and Primer3 (Whitehead Institute for Biomedical Research, Cambridge, MA, USA). Sequence analysis and data handling were carried out using LaserGene, UGENE (Unipro, Novosibirsk, Russia), MEGA7 (Molecular Evolutionary Genetics Analysis; The Pennsylvania State University, PA, USA), and EMBOSS EMBOSS (European Molecular Biology Open Software Suite; EMBnet/EMBL-EBI, Europe).

## 3. Results

### 3.1. AfldrnA Shares Structural Similarity with Other Ascomycetes

The *AfldrnA* gene (locus AFL2G_04716) encodes a basic helix–loop–helix (bHLH) transcription factor. The open reading frame (ORF) of *AfldrnA* is 1200 bp in length, comprising a 1146 bp coding sequence interrupted by a single 54 bp intron located near the 5′ end and flanked by *Ear*I and *Mwo*I restriction sites (Figure S2A). The predicted AFLDrnA protein consists of 382 amino acids. BLASTP analysis revealed homologous proteins in *A. oryzae*, *A. fumigatus*, *A. nidulans*, and other related species (Figure S2B), indicating strong conservation among Ascomycetes.

### 3.2. Construction and Confirmation of the AfldrnA Deletion Mutant

The *AfldrnA* gene was deleted via *A. fumigatus pyrG*-mediated gene replacement, as described in the Materials and Methods section. PCR screening using the primer pairs *AfldrnA* 5′ nest and *AfldrnA* 3′ Reverse confirmed gene deletion: the wild type produced a 2.7 kb band, whereas the Δ*AfldrnA* mutant yielded a 3.2 kb product (Figure 1b). Subsequent *Sac*I digestion of the PCR products produced fragments of 2.2 kb and 0.9 kb for the mutant and 1.6 kb and 1.2 kb for the wild type (Figure 1c).

Southern blot hybridization further validated the deletion. Genomic DNA double-digested with *Hind*III and *Xma*I and hybridized with a 0.7 kb DIG-labeled probe revealed a 1.9 kb fragment in the wild type and a 1.5 kb fragment in the Δ*AfldrnA* mutant (Figure 1d). Together, these analyses confirmed successful *AfldrnA* deletion through targeted *pyrG*-mediated gene replacement.

### 3.3. Phenotypic Characterization of the AfldrnA-Deletion Mutant

**Figure 2:**
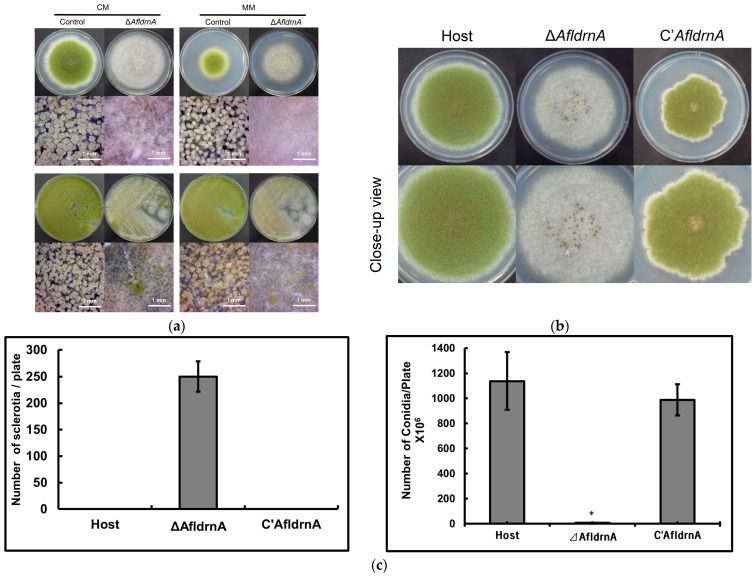
Phenotypic characteristics of the *AfldrnA* deletion mutant. (**a**) Control and Δ*AfldrnA* strains were point-inoculated or streaked onto CM and MM media and incubated at 30 °C for five days. Conidial heads’ images were captured using a DIMIS-M 50× microscope. (**b**) Sclerotia production by Δ*AfldrnA*. Host, Δ*AfldrnA* and complemented (*C*′*AfldrnA*) strains were point-inoculated onto MMU plates and incubated at 30 °C for nine days. The lower row shows magnified views of the upper images. (**c**) Quantitative comparison of sclerotial and conidial counts per plate. Data is presented as mean ± standard deviation. * Indicates a statistically significant difference compared with the other strains (*p* < 0.05).

Phenotypic comparison on CM and MM media (with or without uracil and uridine) revealed distinct developmental differences. The Δ*AfldrnA* mutant formed white colonies with sparse conidiation but exhibited markedly increased sclerotia production compared with the parental strain (Figure 2a–c). Notably, conidiation improved when cultures were streaked rather than point-inoculated.

**Figure 3:**
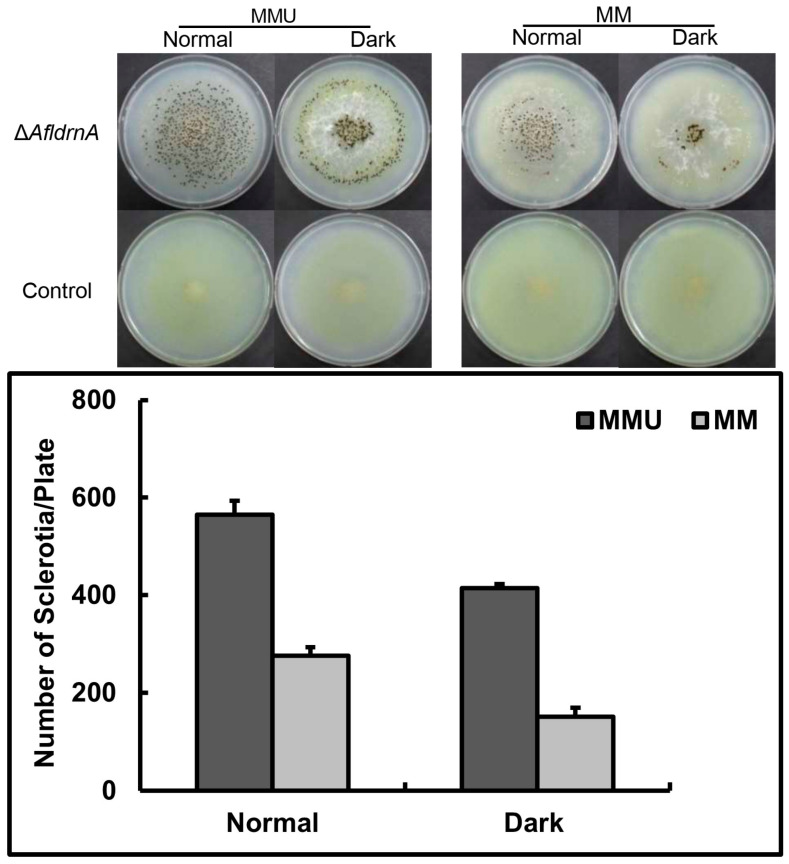
Effect of dark incubation on the sclerotia production in *AfldrnA* deletion mutant. Δ*AfldrnA* cultures were point-inoculated onto MM with or without uracil/uridine (0.12% each) and incubated at 30 °C for nine days under light or dark conditions. The graph shows the average number of sclerotia per plate under the respective conditions.

To assess the influence of darkness on sclerotia production, Δ*AfldrnA* cultures were incubated on MM and MMU (Minimal Medium supplemented with uracil and uridine) at 30 °C for nine days in the dark. Darkness suppressed sclerotia production and promoted hyphal elongation, resulting in cotton-like colonies characterized by rhythmic sclerotial–hyphal growth. Media supplementation with uracil and uridine enhanced the sclerotia yield (Figure 3).

Osmotic stress assays using varying concentrations of potassium chloride showed that the mutant was unable to restore conidiation to the control strain’s levels (Figure S3). Colonies lacked central conidial zones, consistent with the non-conidial phenotypes reported in *drnA* ortholog deletions of *A. fumigatus* [17].

**Figure 4:**
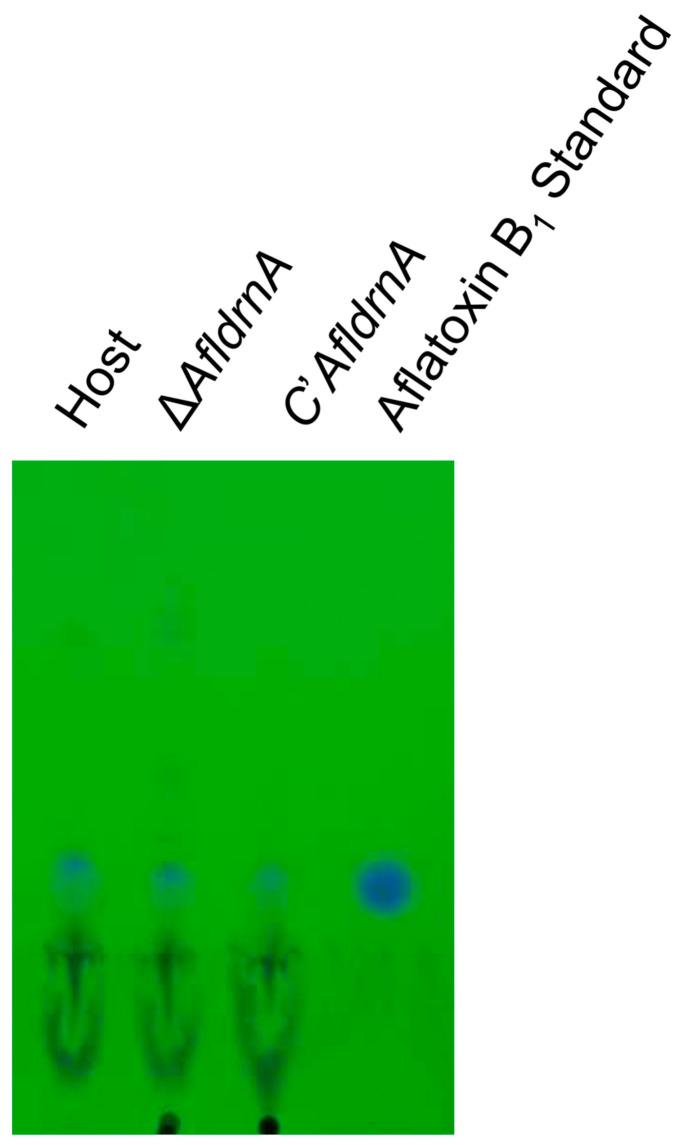
Thin-Layer Chromatography (TLC) analysis of aflatoxin B_1_ production in the Δ*AfldrnA* mutant. Strains were point-inoculated onto potato dextrose agar (PDA) supplemented with uracil/uridine and incubated at 30 °C for 7 days. Following incubation, aflatoxin B_1_ was extracted and analyzed by TLC to evaluate aflatoxin B_1_ production.

To test the role of *AfldrnA* in secondary metabolism, aflatoxin B_1_ production was analyzed by Thin-Layer Chromatography (TLC) under UV light. The Δ*AfldrnA* mutant produced aflatoxin B_1_ levels comparable to the control strain (Figure 4), indicating that *AfldrnA* is not essential for aflatoxin B_1_ biosynthesis.

Antifungal susceptibility testing using Multodisc YEASTS (Liofilchem, Cat. No. 95280, Roseto degli Abruzzi, Italy) and E-test strips revealed no significant differences in sensitivity between the Δ*AfldrnA* mutant and the control strains (Figure S4). Growth assays showed comparable radial expansion on CM and MM media after four days at 30 °C, indicating that *AfldrnA* deletion did not significantly affect vegetative growth (Figure S4).

### 3.4. Construction and Characterization of AfldrnA-Complemented Strain

**Figure 5:**
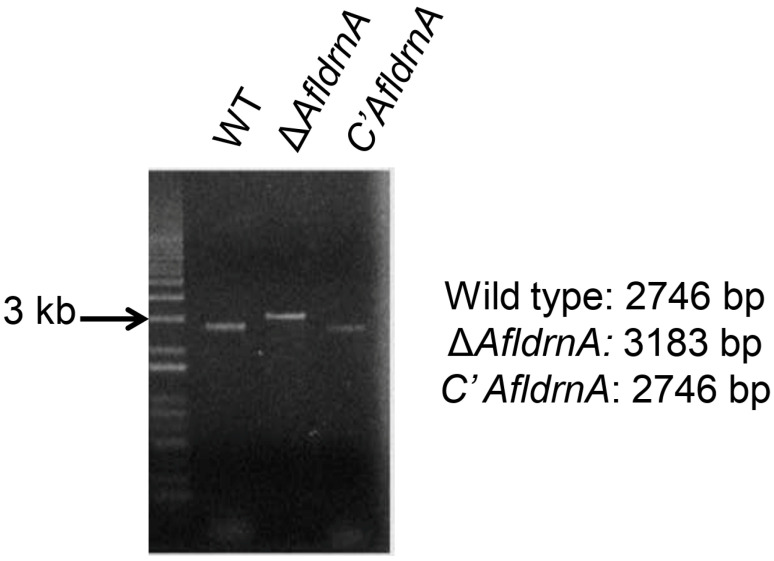
Screening of *AfldrnA*-complemented strains by PCR. Genomic DNA was extracted from pyrithiamine-resistant transformant and subjected to PCR using *AfldrnA* 5′nest and *AfldrnA* 3′ Reverse primers. A 2.7 kb fragment was amplified in the control and complemented strains, while the deletion strain yielded a 3.2 kb.

Complementation of the Δ*AfldrnA* mutant was performed using a plasmid containing *AfldrnA* and the pyrithiamine resistance gene (*ptrA*) as a selectable marker. PCR verification with *AfldrnA* 5′ nest and *AfldrnA* 3′ Reverse primers detected a 2.7 kb product in the complemented and control strains, whereas the deletion mutant yielded a 3.2 kb fragment (Figure 5).

Growth assays on media containing 0.1 µg/mL or 0.3 µg/mL pyrithiamine, with or without uracil and uridine supplementation, showed that the complemented strain grew only on uracil and uridine-supplemented media, irrespective of pyrithiamine presence. This suggests the possible loss of the *pyrG* marker and reversion to uracil auxotrophy. The observed pyrithiamine resistance may reflect integration of two plasmid copies, one restoring *AfldrnA* and one conferring resistance.

**Figure 6:**
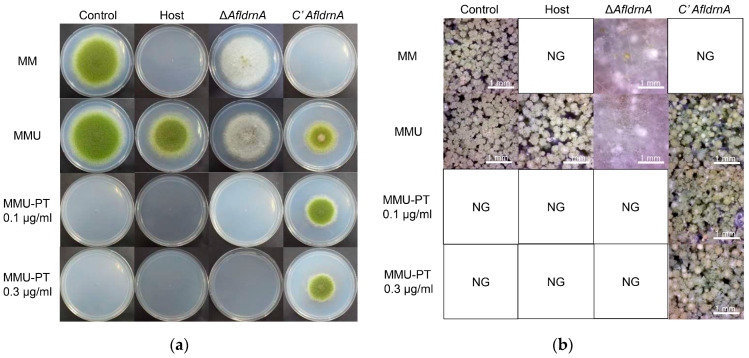
Phenotype of the *AfldrnA*-complemented strain (*C′AfldrnA*). (**a**) The Control, Host, Δ*AfldrnA*, and complemented strains were point-inoculated onto MM, MMU, MMU supplemented with pyrithiamine (0.1 µg/mL), and MMU with 0.3 µg/mL pyrithiamine. Cultures were incubated at 30 °C for six days. (**b**) Stereomicroscopic observation at 50× magnification (DIMIS-M microscope) of image (**a**). NG stands for “No Growth”.

Phenotypically, complementation restored normal conidiation and significantly reduced sclerotia production. Only a single sclerotium was observed on MM supplemented with uracil, uridine, and 0.1 µg/mL pyrithiamine (Figure 6). These findings confirm that the loss of *AfldrnA* caused the aconidial phenotype and excessive sclerotia development.

## 4. Discussion

The helix–loop–helix (HLH) family of transcription factors is essential for regulating diverse fungal developmental processes, including penicillin biosynthesis, asexual and sexual development, cell cycle progression, and other cellular and physiological functions [25]. Asexual sporulation, the predominant reproductive strategy among filamentous fungi, entails the production of conidia through mitotic division. Although the precise mechanisms initiating asexual spore formation remain incompletely understood, the central regulatory network governing conidiation has been well characterized in *Aspergillus nidulans*. Key developmental regulators, including *brlA, abaA, and wetA*, orchestrate conidiophore development and spore maturation [1,26,27,28]. In *A. nidulans*, the *drnA* ortholog, which functions under the regulation of *nsdD*, plays a pivotal role in maintaining the balance between asexual and sexual development—its deletion suppresses conidiation, while overexpression inhibits sexual reproduction [18]. Comparable regulatory interactions have been observed in *A. oryzae*, where disruption of *ecdR*, an orthologous gene, impairs conidiation and enhances sclerotia production [25,29], and in *A. fumigatus*, where *AfudrnA*-deleted strains exhibit similar non-conidiated phenotype [17].

In *A. flavus*, deletion of the *drnA* gene (*AfldrnA*) led to a marked loss of conidiation accompanied by enhanced sclerotia production, mirroring the developmental phenotypes reported in *A. oryzae*, *A. nidulans*, and *A. fumigatus*. Complementation of the Δ*AfldrnA* mutant with the intact *AfldrnA* gene restored normal conidiation and reduced sclerotia production, confirming that the observed phenotypes were directly attributable to the loss of *AfldrnA*. These findings reinforce the conserved regulatory function of *drnA* orthologs across *Aspergillus* species in maintaining developmental equilibrium between conidiation and sclerotia or sexual differentiation.

Upstream of this pathway, conidiation in *A. flavus* is modulated by activators such as *fluG*, which triggers the developmental cascade that leads to *brlA* activation [10,30]. In *A. nidulans*, *fluG* deletion mutants display a fluffy, aconidial phenotype consisting of undifferentiated vegetative hyphae [30,31]. In *A. flavus*, *fluG* disruption delays conidiation and enhances sclerotia formation, particularly under dark conditions [16]. However, the Δ*AfldrnA* mutant exhibited increased hyphal elongation and reduced sclerotial formation when incubated in the dark, suggesting an opposite regulatory influence of *AfldrnA* in light-dependent development. These findings may indicate a possible direct or indirect interaction between *AfldrnA* and the *fluG*-mediated pathway in coordinating the balance between asexual and sclerotia development in *A. flavus*.

Interestingly, deletion of *AfldrnA* did not affect aflatoxin B_1_ production, aligning with previous findings in *A. flavus* where *fluG*-null mutants retained aflatoxin production [16]. In contrast, *A. nidulans fluG* mutants fail to produce sterigmatocystin, the aflatoxin precursor [32]. These results indicate that although *AfldrnA* and *fluG* are central regulators of conidiation and sclerotia development, their influence on secondary metabolism in *A. flavus* is limited. Nevertheless, further investigation is warranted to determine whether *AfldrnA* exerts indirect regulatory effects on secondary metabolic pathways.

A subtle increase in conidiation was observed when the Δ*AfldrnA* mutant was streaked on solid media rather than point-inoculated. This enhancement may result from improved nutrient diffusion and oxygen availability along the streaked surface, which could promote the activation of conidiation-related signaling cascades. However, conidial production remained significantly below that of the control strain, confirming *AfldrnA’s* essential role in sporulation.

Exposure of the Δ*AfldrnA* mutant to osmotic stress via potassium chloride supplementation did not restore conidiation, indicating that *AfldrnA* is unlikely to participate in osmotic stress signaling pathways. Likewise, antifungal susceptibility assays revealed no notable differences between Δ*AfldrnA* and control strains, suggesting that *AfldrnA* does not contribute to antifungal resistance or sensitivity mechanisms. Moreover, comparable colony growth rates between mutant and control strains imply that *AfldrnA* primarily regulates developmental differentiation rather than vegetative growth.

Complementation experiments further substantiated the functional role of *AfldrnA*. Reintroduction of the gene restored conidiation and abolished excessive sclerotia production, demonstrating that the phenotypic alterations in the deletion mutant were specifically caused by the absence of *AfldrnA*. Collectively, these findings establish *AfldrnA* as a key developmental regulator in *A. flavus*, controlling the balance between conidiation and sclerotia reproduction without significantly impacting secondary metabolism or stress response pathways.

## 5. Conclusions

This study demonstrates the pivotal role of the *AfldrnA* gene in regulating asexual conidiation and sclerotia production in *A. flavus*. Deletion of *AfldrnA* resulted in severe impairment of conidiation and enhanced sclerotia production, whereas complementation with the wild-type gene restored normal sporulation and suppressed sclerotia production. These findings are consistent with the functions of *drnA* orthologs in other *Aspergillus* species, underscoring its evolutionary conservation as a developmental regulator. Notably, *AfldrnA* deletion did not alter aflatoxin B_1_ biosynthesis, antifungal susceptibility, or growth rate, suggesting that its regulatory influence is confined primarily to morphological differentiation. Overall, *AfldrnA* emerges as a critical determinant of fungal reproductive balance, offering new insights into the genetic control of conidiation and sclerotial morphogenesis in *A. flavus*.

## Figures and Tables

**Table 1:** List of the used strains in the study.

Strain	Genotype	Source
NRRL3357	Wild type	Wild type
NRRL3357.5	*pyrG^−^*	Host
NRRL3357.10	*pyrG^−^::pyrG*	Control [This study]
MOH-12	*pyrG^−^;* ∆*AfldrnA::AfpyrG*	This study
Sud-1	*pyrG^−^;* ∆*drnA::AfpyrG; drnA^+^::ptrA::*∆*AfpyrG*	This study

**Table 2:** The list of the used oligonucleotides.

Name	Sequence
*AfldrnA* 5′ For	AAAAGCGAAGATAGAAAATGTT
*AfldrnA* 5′ Rev *pyrG* tail	GGTGAAGAGCATTGTTTGAGGCATATGTATGTGCGAATGTCTGA
*AfldrnA* 3′ For *pyrG* tail	AGTGCCTCCTCTCAGACAGAATTTCGAACGGGACTGATTATTGA
*AfldrnA* 3′ Rev	ACCTTTCCCCATGTTGATTTCT
*AfldrnA* 5′ nest	AAAAGAAAAACCCAAATAATAC
*AfldrnA* 3′ nest	AAGCCTTCATCATGTCGGTAGT
*AfldrnA* complementary For-*Asc*I	GGAGAAGGCGCGCC GCGGAAGGTTGAGGCTACTT
*AfldrnA* complementary Rev-*Sma*I	GGAGAACCCGGG GTTCGCCAGGATCCAGTGAA
*AfldrnA* Comp.Frag.Seq. A Rev	TCCGACAATGACATACAGATCTC
*AfldrnA* Comp.Frag.Seq. B For	ACTATTGATCGATCTCTGAACCT
*AfldrnA* Comp.Frag.Seq. B Rev	GTCTCAGGGCGGGAAAGAC
*AfldrnA* Comp.Frag.Seq. C For	CTCCCATAGCAACATCCTCCC
*AfldrnA* Comp.Frag.Seq. C Rev	GCGAAGATCTTGGAGGCCA
*AfldrnA* Comp.Frag.Seq. D For	CAACGCTCCTAACGCAACAG
*AfldrnA* Comp.Frag.Seq. D Rev	GATTCCACCCGGCCCGTTC
*AfldrnA* Comp.Frag.Seq. E For	GTAAGGGGAGCATGGTCACG
*AfldrnA* Comp.Frag.Seq. E Rev	AAAACACGTAGGCCAGGCAA
*AfldrnA* Comp.Frag.Seq. F For	CCCAGCCTCTTACCCAGTTT

The underlined sequences correspond to the underlined names indicated in the oligonucleotides’ name column.

## Data Availability

All data generated or analyzed during this study are included in this published article and its Supplementary Materials. Additional datasets related to this work are available from the corresponding author upon request.

## Supplementary Materials

The following supporting information can be downloaded at: https://www.mdpi.com/article/10.3390/jof12040277/s1. Figure S1: Schematic representation of the *AfldrnA* complementation vector construction; Figure S2: Schematic representation of the *AfldrnA* gene nucleotide sequence and Phylogenetic tree demonstrating the evolutionary relationships of DrnA protein; Figure S3: Effect of Potassium Chloride-induced osmotic stress on Δ*AfldrnA*; Figure S4: Antifungal Sensitivity Test of the Δ*AfldrnA* Strain; Figure S5: Growth rate of the *AfldrnA* deletion mutant.
