# Supplementary material for: The AfldrnA Transcription Factor Is a Pivotal Regulator of the Conidiation–Sclerotial Formation Balance in Aspergillus flavus
Source: J Fungi (Basel). 2026 Apr 14;12(4):277. doi: 10.3390/jof12040277 (PMC13117240; doi:10.3390/jof12040277)
Supplement: Supplementary file 1 [file jof-12-00277-s001.zip › jof-4230180-supplementary.pdf]

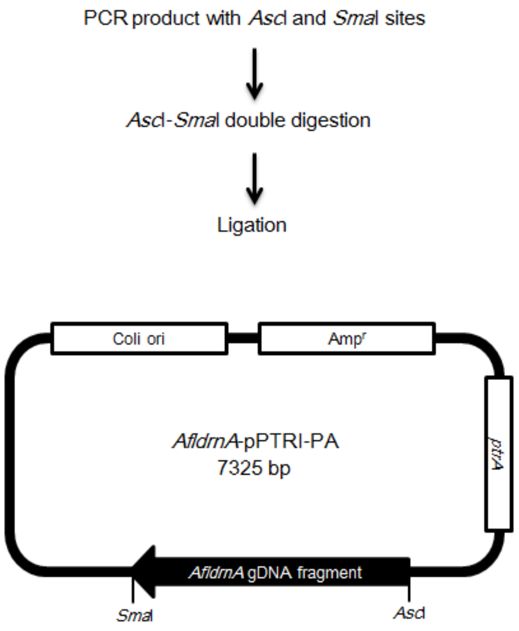

Figure S1: Schematic representation of the *AfldrnA* complementation vector construction. A 2.5 kb genomic DNA fragment, which includes a 1.2 kb *AfldrnA* ORF, a 1.1 kb upstream sequence, and a 0.2 kb downstream sequence, was amplified using the primer pairs *AfldrnA* Complementary For-*Asc*I and *AfldrnA* Complementary Rev-*Sma*I. The resulting PCR product was digested with *Asc*I and *Sma*I and subsequently ligated into the pPTRI-PA vector at the corresponding enzyme sites. The final construct contains the *AfldrnA* genomic DNA fragment along with the pyrithiamine resistance gene, *ptrA*.

A

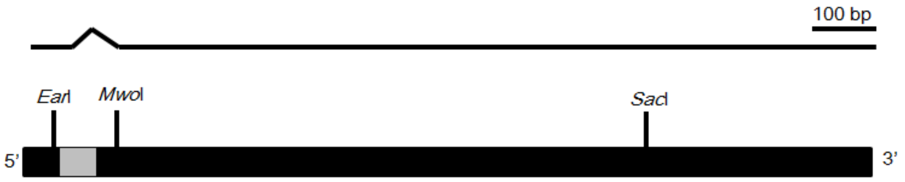

B

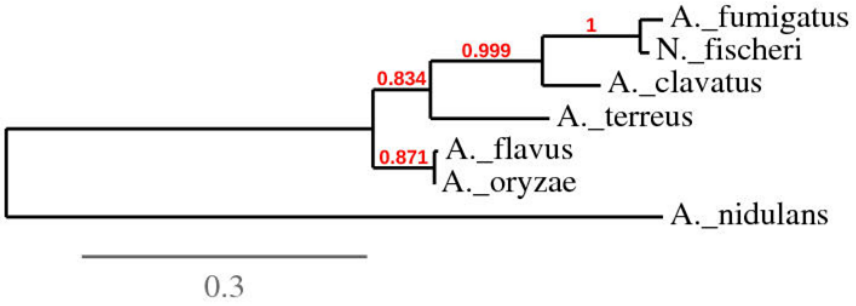

Figure S2. (A) Schematic representation of the *AfldrnA* gene nucleotide sequence, showing a 1200-nucleotide ORF composed of three regions (from 5' to 3'): a 44-nucleotide coding sequenc e, followed by a 54-nucleotide intron (gray box), and a 1102-nucleotide coding sequence. The int ron is flanked by *Ear*I and *Mwo*I restriction sites, while the long coding region is interrupted by a *Sac*I site. **(B)** Phylogenetic tree demonstrating the evolutionary relationships of DrnA proteins ac ross various Ascomycete species, highlighting their sequence similarities.

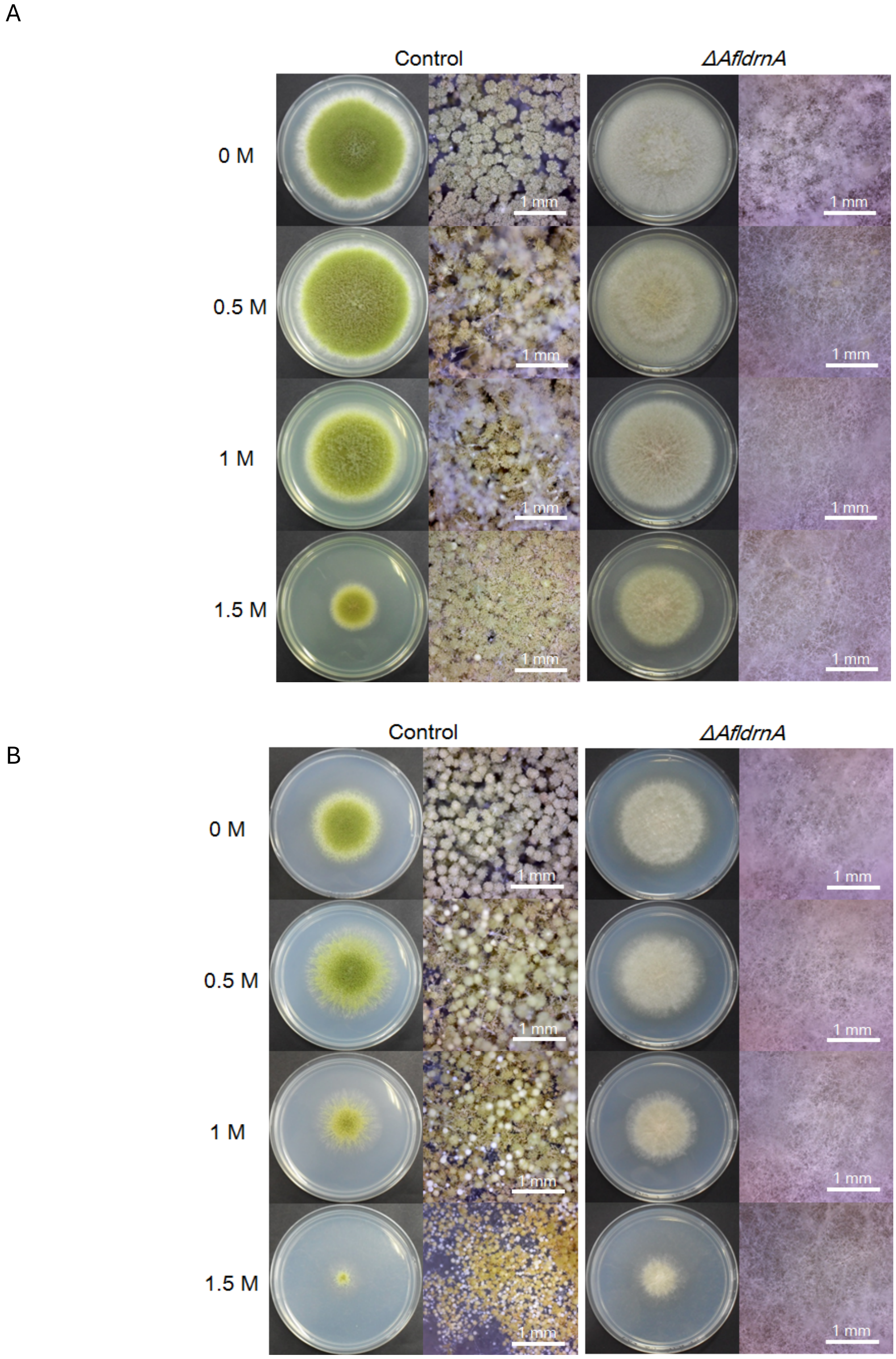
A
Control
ΔAfldrnA
0 M
0.5 M
1 M
1.5 M
1 mm
B
Control
ΔAfldrnA
0 M
0.5 M
1 M
1.5 M
1 mm

Figure S3: Effect of Potassium Chloride-induced osmotic stress on Δ*AfldrnA*.

The phenotype of the Δ*AfldrnA* mutant was assessed under conditions promoting asexual develop ment using different concentrations of KCl. **(A)** Growth of the Δ*AfldrnA* mutant on CM media wi th varying KCl concentrations. **(B)** Growth of the Δ*AfldrnA* mutant on MM media with varying K Cl concentrations. All strains were incubated at 30°C for 5 days, and 50X magnification images w ere captured using a DIMIS-M microscope to observe the effects of osmotic stress.

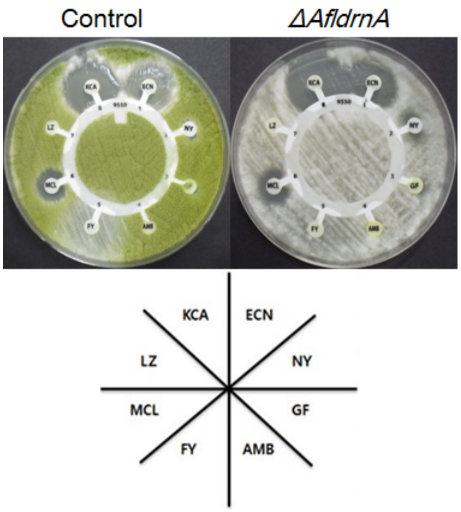

		Multodisc-YEASTS	
1	ECN	ECONAZOLE	10 μg
2	NY	NYSTATIN	100 I.U.
3	GF	GRISEOFULVIN	10 μg
4	AMB	AMPHOTERICIN B	20 μg
5	FY	FLUCYTOSINE	1 μg
6	MCL	MICONAZOLE	10 μg
7	LZ	METRONIDAZOLE	10 μg
8	KCA	KETOCONAZOLE	10 μg

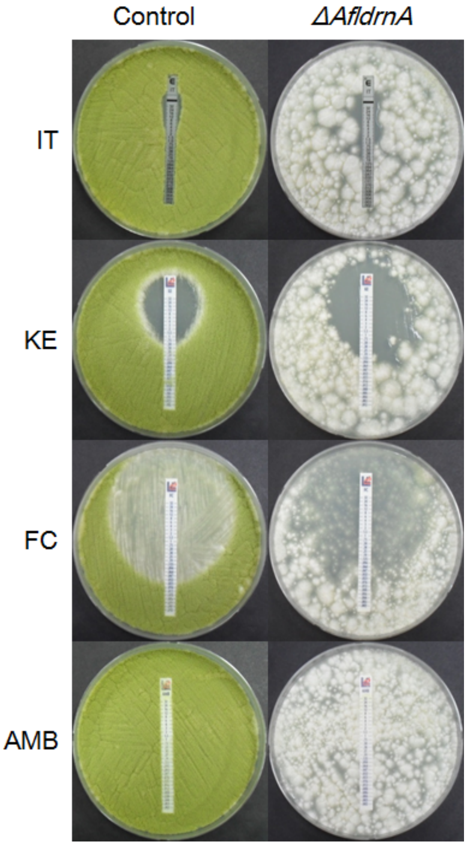

Figure S4: Antifungal Sensitivity Test of the Δ*AfldrnA* Strain. (A) Multodisc Yeast sensitivity test using the Multodisc YEASTS (Liofilchem, Italy). **(B)** E-test method for antifungal sensitivity. All strains were streaked onto CM media and incubated at 30°C for 48 hours to assess their sensitivity to antifungal agents.

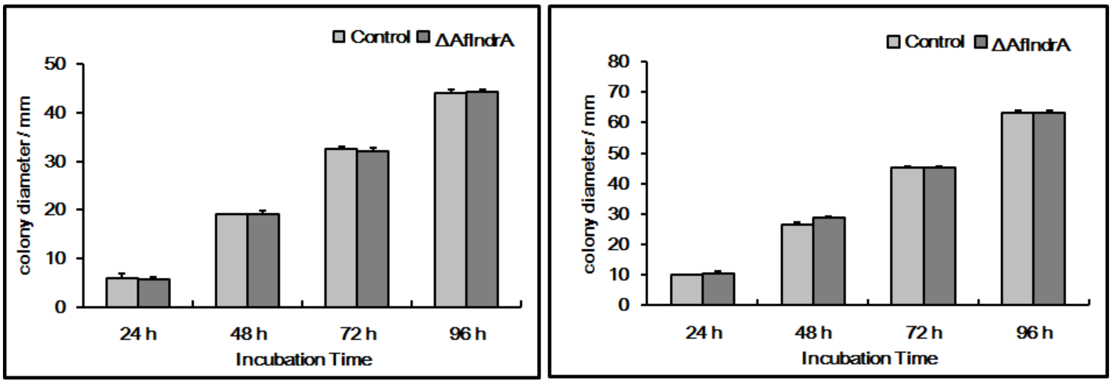

Figure S5: Growth rate of the *AfldrnA* deletion mutant. The strains were point-inoculated onto MM (left chart) and CM (right chart) plates and incubated at 30°C for 4 days. The growth rates were measured daily by recording the colony diameters. The X-axis represents the incubation time in days, and the Y-axis represents the colony diameter in millimeters.
